# Mobile Apps for Tinnitus: Systematic Search in App Stores and Review of Intervention Components and Behavior Change Techniques

**DOI:** 10.2196/66151

**Published:** 2026-05-19

**Authors:** Alina Rinn, Sarah Goetsch, Sandy Hannibal, Dirk Lehr, Cornelia Weise

**Affiliations:** 1 Clinical Psychology and Psychotherapy, Philipps-University of Marburg, Gutenbergstraße 18, Marburg, Germany, 49 6421 28 22839; 2 Department of Health Psychology and Applied Biological Psychology, Leuphana University Lüneburg, Lüneburg, Germany; 3 Clinical Psychology and Behavioral Health Technology, Dept. of Psychology, Friedrich-Alexander-Universität Erlangen-Nürnberg, Erlangen, Germany

**Keywords:** tinnitus, smartphone apps, behavior change techniques, intervention components, internet interventions

## Abstract

**Background:**

Previous research suggests that 14.4% of the general population is affected by tinnitus. For some of those affected, the ear noise is bothersome or associated with severe distress. There are various treatment options such as cognitive behavioral therapy (CBT), sound therapy, or hearing aids. In addition to browser-based online interventions, mobile apps have been introduced as novel treatment approaches. Previous studies have identified several apps aimed at supporting users with tinnitus. Yet, knowledge about the content of tinnitus apps is limited.

**Objective:**

This study aimed to provide an overview of apps specifically developed for tinnitus by analyzing general app characteristics, as well as app content, focusing on intervention components and behavior change techniques (BCTs).

**Methods:**

A systematic search using 7 search terms (eg, tinnitus and ear noise) was conducted in the Google Play Store and the Apple App Store. Apps designed specifically for tinnitus and available in German or English met the inclusion criteria. Two independent trained raters assessed general app characteristics (eg, age group and costs) using the app description section of the German version of the Mobile App Rating Scale. In addition, raters analyzed app content using the BCT taxonomy (v1) and a list of typical intervention components in tinnitus treatment. Differences in ratings were discussed, and a third trained rater was consulted if no consensus was reached.

**Results:**

A total of 1198 apps were identified in the systematic search. Of those, 69 apps were included in the final analysis. Fifty-two apps were available for free, 23 of which offered in-app purchases. Among the 17 paid apps, costs ranged between €0.69 (US $0.81) and €450 (US $527) per 12 months. Fifty-eight of 69 apps provided sounds (eg, white noise and nature sounds). Many apps assessed tinnitus characteristics (n=38) and provided information about tinnitus (n=27). The most frequently used BCTs were “instruction on how to perform the behavior” (n=25; eg, audio instructions for relaxation techniques), “feedback on behavior” (n=11), “behavioral practice/rehearsal” (n=11), “information about health consequences” (n=11), “information about emotional consequences” (n=11), and “prompts/cues” (n=11). The number of BCTs implemented varied widely across apps (0‐18 per app).

**Conclusions:**

Most tinnitus apps offer sound-based interventions (eg, white noise and nature sounds). Notably, CBT elements (eg, cognitive restructuring, attention training, and relaxation training) are implemented less frequently, despite CBT being recommended in tinnitus treatment guidelines. Further research on the efficacy of tinnitus apps is needed. Transparent reporting of intervention techniques may help clarify mechanisms of action and support the replication of effective interventions. Given the large number of readily accessible apps, this study provides an overview relevant to both researchers and health care professionals.

## Introduction

Tinnitus is often described as a hissing, sizzling, or ringing in one or both ears or within the head and is defined as an auditory perception without corresponding external sound [1]. Recent meta-analytic findings point to a pooled prevalence of 14.4% for any tinnitus in adults [2]. For a smaller proportion, the ear noise is bothersome or related to severe distress. For example, 1.2% of the participants in a European Tinnitus Survey reported severe tinnitus [3,4]. Sleep disturbances and concentration difficulties, as well as depression and anxiety, can be associated with the ear noise [5-7].

Both patients seeking treatment and practitioners encounter numerous options with a varying base of evidence (eg, medication, hearing aids, cognitive behavioral therapy, or sound therapy) [1,8]. Digital innovation has paved the way for new methods to deliver treatment by providing internet- and mobile-based interventions (IMIs) [9]. IMIs have the potential to provide access to evidence-based interventions on a larger scale. They may accompany traditional interventions, potentially enhancing their effects or reducing costs [10]. Nevertheless, questions and potential risks arise when implementing IMIs, for example, concerning data security or the efficacy of stand-alone smartphone apps [10-12].

In recent years, especially internet-based interventions that provide cognitive behavioral strategies to people affected by tinnitus have been developed. These interventions target the reduction of tinnitus distress and have shown promising effects in several studies [9,13]. In addition to these web-based interventions, a range of smartphone apps aimed at alleviating burdensome tinnitus are easily accessible on app stores [14,15]. An understanding of what is being offered in these interventions may foster the replication of effective interventions, help identify potential mechanisms of action, and inform future intervention development [16]. However, to date, information and studies on the content and aims of tinnitus apps remain scarce.

A primary approach to providing an overview of app content is to categorize the intervention components used (eg, sounds, relaxation training, and information on tinnitus). Previous reviews of tinnitus apps have mostly focused on providing a brief summary on app content, for example, by sorting apps into categories (eg, “tinnitus education, awareness, and prevention,” “tinnitus relief,” “sound therapy,” or “individual therapy”) [14,15,17]. Two of these reviews additionally provide brief descriptions of what individual apps offer, without necessarily distinguishing the specific intervention components implemented (eg, offering sounds to mask tinnitus, providing individual therapy for tinnitus) [15,17]. Sereda et al [18] provided a detailed description of intervention components and functions of apps. However, the group did not systematically search the app stores but rather analyzed some of the apps that people affected by tinnitus reported using in their daily life [18]. We wanted to add to this line of research by using a systematic approach to differentiate individual intervention components (as described in treatment guidelines or handbooks on tinnitus therapy), thereby providing a detailed overview of the intervention components used.

A second approach for categorizing app content is to identify the behavior change techniques (BCTs) used [19]. Michie et al [19,20] developed a cross-domain taxonomy that includes 93 BCTs (eg, “self-monitoring of behavior”), allocated to 16 categories (eg, “feedback and monitoring”). This approach, rooted in health psychology, may be a useful second perspective to understand what might enhance active tinnitus management by fostering health behavior change. Prior studies on tinnitus have documented the BCTs used in an internet-based intervention by applying the behavior change wheel [21]. Furthermore, BCTs, alongside other measures, were assessed to describe the intervention content of a single internet-based intervention and several self-help interventions [16,22]. The authors concluded that intervention coding fostered the understanding of potential mechanisms of action and may be used to guide future developments [16,21,22]. BCTs have not yet been investigated in tinnitus apps.

Taken together, our study focused on the following research questions:

- How many German and English apps specifically developed for tinnitus are available on the Google Play Store and the Apple App Store?
- Which intervention components are provided in tinnitus apps?
- Which BCTs are used in tinnitus apps?

Findings on the quality and content of a subsample of the apps included in this study, namely 21 German apps, have previously been published in German to contribute to ongoing discussions specific to the German health care system [23]. Based on an updated systematic search, we aim to provide a broad overview of current app content in both English and German apps. To advance tinnitus-specific research and intervention development, and to inform researchers, practitioners, and future tinnitus app development, we focused exclusively on apps explicitly developed for people affected by tinnitus.

## Methods

### Systematic Search

The project was registered at the Open Science Framework [24]. The German Google Play Store and Apple App Store (iTunes) were searched to identify both paid and free Android and iOS apps. The following search terms, as well as their German equivalents, were used: “tinnitus,” “ear ringing,” “ear noise,” “ear buzzing,” “Ohrenklingeln,” “Ohrgeräusch,” and “Ohrensausen.” The search was performed by one of the authors (AR) from November 2020 to April 2021 and updated in February 2023 (conducted by MK and AR). The search strategy was designed to prioritize sensitivity over specificity to ensure a comprehensive capture of all potentially relevant apps. As our aim was to provide an overview of apps explicitly developed for tinnitus, we established strict inclusion and exclusion criteria detailed below to ensure the quality of the final sample.

### Inclusion and Exclusion Criteria

In the first step, identified apps were screened based on information provided in the app store (title, description, user comments, and screenshots). The following inclusion criteria were used: specifically developed for tinnitus and available in German or English. Apps were excluded if one of the following criteria applied: (1) duplicate; (2) app no longer available on the app store; (3) apps with limited functions with a full version of the app being included; (4) app bundles, that is, a group of apps offered together; and (5) app not developed for smartphones but for tablets. If an app was available on both stores, the iOS version was included. App screening for the initial search was performed by one of the authors (1073 apps, AR). In cases where the app screening was inconclusive, a second rater was involved (91 apps, LL; screening for updated search, 125 apps, by AR and MK). In the second step, the remaining apps were tested for eligibility by AR. For this purpose, apps were downloaded and tested again for inclusion criteria. Apps were excluded if either criteria (1) or (2) or one of the following criteria applied: (6) analysis not feasible due to technical shortcomings, (7) app has no app-specific functionality (eg, solely offers eBooks or articles), (8) app in development or test phase only, (9) no access to the app (eg, no purchase via the app store feasible, no user account provided by the app developer upon request), and (10) app offers no intervention itself but accompanies a therapy via earpiece.

### Measures

#### General Characteristics

General app characteristics were assessed using the app description section of the German version of the Mobile App Rating Scale (MARS-G) [25]. We slightly adapted this section for the purpose of our study. The following factors were assessed: platform, cost, affiliations, age group, category in the app store, technical aspects of the app, embedding into routine care, type of use, type of treatment, support, certification, data safety, and barrier-free.

#### Intervention Components

To categorize app content, a list of typical intervention components in tinnitus treatment was developed by the authors (AR, DL, and CW). This list was built based on studies on the components of interventions for tinnitus [26], the German treatment guideline for chronic tinnitus [27], reviews on tinnitus treatment [9,28,29], as well as a handbook on cognitive behavioral therapy for tinnitus [30]. Furthermore, the evidence-based health information guideline was used [31]. The guideline, among other aspects, names criteria on how patients should be informed prior to the start of an intervention. The component “information on the intervention” was developed to capture several of the criteria described. For the list of typical intervention components in tinnitus treatment used to categorize app content, see Multimedia Appendix 1. Moreover, raters analyzed which of the identified intervention components were a key component in the app’s concept (ie, important messages or key skills that are trained when using the app).

#### Behavior Change Techniques

To assess which techniques were used to encourage changes in behavior, the BCT taxonomy (v1) [19] was used. This taxonomy includes 93 techniques that might be used in behavior change interventions (eg, “self-monitoring of behavior” or “action planning”). These are allocated to 16 categories (eg, “feedback and monitoring” or “goals and planning”). For each app, raters analyzed which of the 93 techniques described in the BCT taxonomy (v1) were implemented.

### Rater Training

Five raters with a bachelor’s or master’s degree in psychology were trained prior to the app ratings. The training consisted of several steps. First, the raters watched a video introducing the German version of the Mobile App Rating Scale rating, as provided by the authors [25]. As this video contains an example of how to rate a tinnitus app, this specific part was excluded, and instead, the raters viewed parts of the training video created by the authors of the original scale (which included an example of how to rate an app) [32]. Second, the raters used exercises offered on the BCT Taxonomy v1 online platform to train categorizing BCTs (all raters: “Practices”; rater 1: online-training) [33]. Third, to become familiar with the taxonomy, the raters developed and jointly discussed examples of how each BCT might be implemented in a tinnitus app. As a final step, the raters independently analyzed 5 apps developed for pain management and subsequently discussed their results until agreement was reached.

### Procedure and Data Analysis

Two raters independently tested each app for at least 20 minutes and then proceeded to rate the app. The full versions of the apps (including all app features) were tested. Each rater was provided with a list of apps, which had to be rated in descending order. The order specified differed between raters to minimize potential order effects. Once all apps were analyzed, raters discussed the results based on differences in the ratings of general characteristics, intervention components, and BCTs. If no consensus was reached, a third rater participated in the discussion. Descriptive statistics (eg, frequencies of individual BCTs) were calculated using IBM SPSS Statistics version 28 [34].

## Results

### Systematic Search

**Figure 1.**
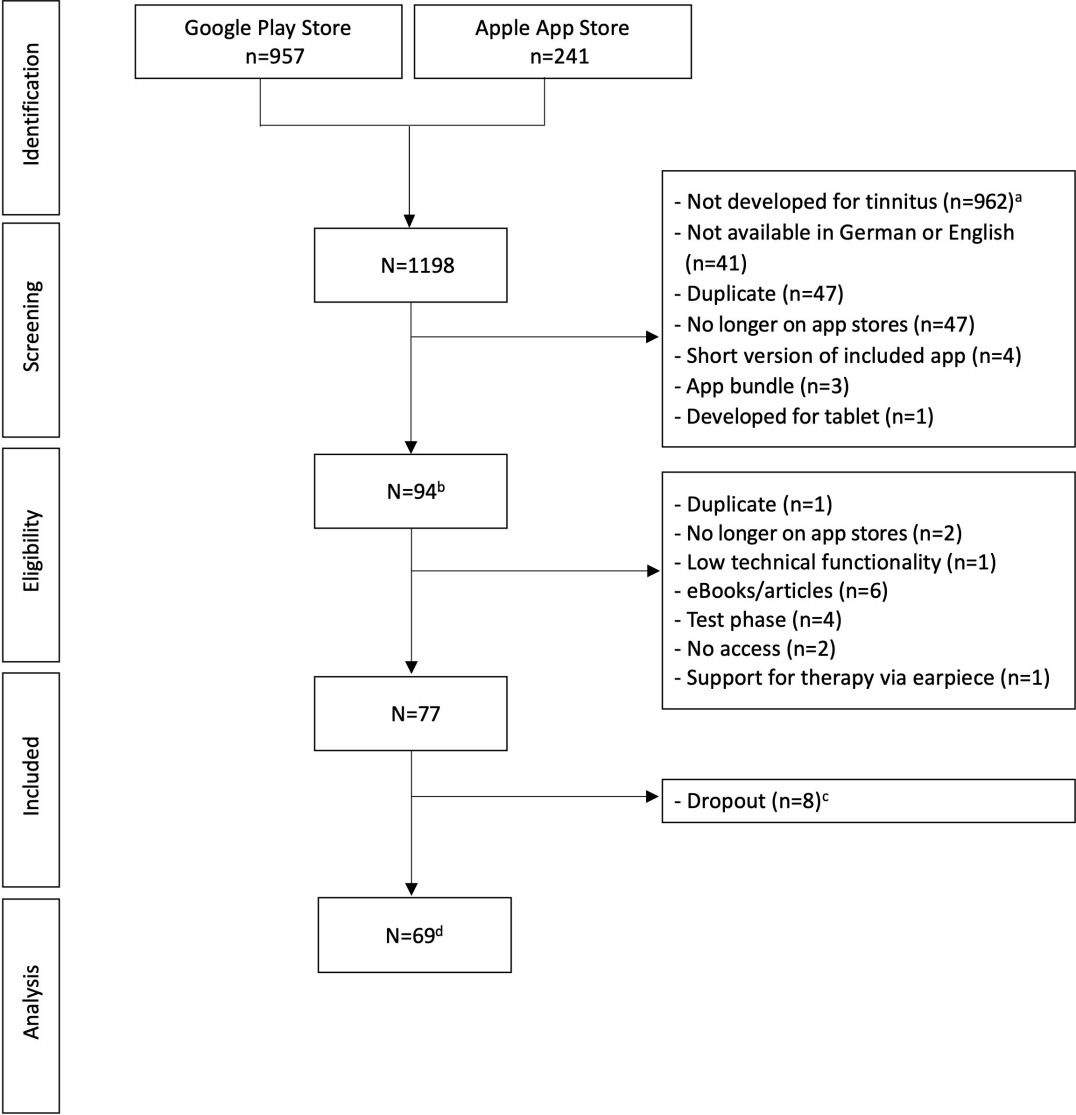
Systematic search and inclusion of tinnitus apps. ^a^809 apps were not developed for tinnitus, for example, apps offering a decibel meter; 153 apps did mention tinnitus but were not explicitly designed for tinnitus: 115 apps offering sounds for sleep, to foster concentration, to accompany meditation or yoga praxis, to reduce stress, for tinnitus; 17 apps for hearing aid devices offering tinnitus programs; 21 apps mentioning tinnitus in the app title or description (eg, information on different diseases of the ear, nose, or throat; frequency generator; and listening to a tinnitus tone). ^b^Inclusion of the full version of an app following a suggestion of the app developer. ^c^Six apps no longer on app stores, 1 app with low technical functionality, 1 link to website, and 1 duplicate. ^d^One app was no longer available on the Apple App Store, but still offered on the Google Play Store, so the Android version was tested instead; one app was tested with limited functions (no full version or access to the app store description available).

The initial search using the 7 search terms mentioned above yielded 1198 apps on the app stores (Google Play Store: n=957; Apple App Store: n=241). Of these, 1105 apps were excluded during screening. Most of the apps (n=962) were excluded as they were not explicitly developed for tinnitus (n=809 addressed topics other than tinnitus, eg, decibel meters; n=153 apps mentioned tinnitus but were not explicitly developed for it, eg, apps accompanying hearing aid devices; Figure 1). The remaining 94 apps were downloaded and tested for eligibility. Of these, 17 apps were excluded (eg, due to low technical functionality), leaving 77 apps that fulfilled all inclusion criteria. During the app testing, a further 8 apps had to be excluded (eg, no longer available on the app stores). Accordingly, 69 apps were included in the final analysis. Figure 1 displays the flowchart of the systematic search and inclusion process for tinnitus apps, and the caption provides further detailed information on the selection process. The list of all apps included in the final analysis can be found in Multimedia Appendix 1.

### App Characteristics

A total of 45 iOS and 24 Android apps were analyzed. Fifty-two apps were free, with 23 of them offering in-app purchases. The price for 17 paid apps ranged between €0.69 (US $0.81) and €450 (US $527) per 12 months. The majority of tinnitus apps were offered in the app store categories medical (n=29 apps) or health and fitness (n=28 apps). For 29 apps, we were not able to identify the developers or providers. Sixty-six apps offered a treatment, with 1 app additionally including content for prevention. One app aimed to track symptoms and 1 app offered an assessment of the tinnitus tone. Three apps had an app community (eg, motivational notifications can be sent to other users), and 3 apps offered an exchange with others via social media or messenger apps. Almost all apps were not by design embedded into routine care (n=63). Only 3 apps allowed for the sharing of information (eg, user statistics) with a health care professional. Few apps provided support for users (n=8), most of them regarding potential technical issues (n=6). For a full overview of app characteristics, see Table 1.

**Table 1.** App characteristics for all apps included^a^.

Characteristics	Values, n (%)
Platform	
iOS	45 (65.2)
Android	24 (34.8)
Cost^b^	
Free	52 (75.4)
In-app purchases available (range: €109 [US $1.28]-€6799 [US $79.59]/mo)	23 (33.3)
Paid (range: €0.69 [US $0.81]-€450 [US $527]/12 mo)	17 (24.6)
Affiliations	
Unknown	29 (42)
Commercial	30 (43.5)
Nonprofit organization	1 (<0.1)
Hospital	1 (<0.1)
*Other*: private individual	5 (0.1)
*Other:* foundation	3 (<0.1)
Age group	
18+ y	1 (<0.1)
USK^c^ rating	24 (34.8)
Age unspecified	1 (<0.1)
*Other*: age rating Apple	44 (63.8)
Category in the app store^d^	
Lifestyle	1 (<0.1)
Medical	29 (42.0)
Health and fitness^e^	28 (40.6)
*Other:* music and audio	5 (0.1)
*Other:* social networking	1 (<0.1)
*Other:* entertainment	1 (<0.1)
*Other:* tools	1 (<0.1)
*Other:* utilities	2 (<0.1)
Technical aspects of app	
Exchange with others (eg, social media)	3 (<0.1)
Has an app community	3 (<0.1)
Needs web access to function	22 (31.9)
*Other:* headphones needed	1 (<0.1)
Embedding into routine care	
None	63 (91.3)
Sharing of content (eg, user statistics) with health care professional	3 (<0.1)
*Other*^f^	4 (0.1)
Type of use	
Prevention	1 (<0.1)
Treatment	66 (95.7)
Unspecified	1 (<0.1)
*Other*^g^	3 (<0.1)
Type of treatment	
Stand-alone	69 (100)
Support	
Guided synchron	1 (<0.1)
Guided asynchron	2 (<0.1)
Technically guided	6 (0.1)
Unguided	61 (88.4)
Certification	
None	61 (88.4)^h^
Accordance with German medical device law	4 (0.1)
*Other:* CE^i^ marked	6 (0.1)
*Other:* certified in accordance with 93/42/EEC for Medical Devices	1 (<0.1)
Data safety	
Allows password protection	13 (18.8)
Requires login	13 (18.8)
Has a privacy policy	53 (76.8)
Barrier-free	
No	69 (100)

a Characteristics of 69 apps as assessed by the app description section Mobile App Rating Scale–German (adapted).

b n=2 cost coverage by German health insurance company.

c USK: German Entertainment Software Self-Regulation Body.

d For 1 app, categories were not assessed, as the app store description was no longer available.

e One app listed “mental well-being” as a second app category.

f n=2 usage primarily within the context of study participation; n=2 further guidance, customized therapy, or online training are offered on the app`s website.

g n=1 tracking symptoms, n=1 assessment of tinnitus tone, and n=1 additionally assessing sleep quality and problematic internet use.

h n=1 developed in accordance with norms IEC 62304 and IEC 82304 as well as the “Good Automated Manufacturing Practice 5” (GAMP 5).

i CE: Conformité Européenne.

### Intervention Components

Intervention components were assessed using a list of components in tinnitus treatment developed by the authors. Most frequently used intervention components were sounds (eg, white noise, nature sounds, and music), which were implemented in 58 of 69 apps. In 52 apps, sounds were a key element. An assessment of tinnitus characteristics was offered by 38 apps (key element in 22 apps). Furthermore, information on tinnitus (n=27, key element in 16 apps) and the provision of information on, or assessment of risk factors for tinnitus (n=23) were implemented in several apps. Less than a third of the apps assessed tinnitus distress (n=18) or provided information on or assessed further otological symptoms (n=16). Further intervention components typically used in CBT were implemented in a smaller number of apps: relaxation training (n=16), techniques to improve sleep (n=12), identification of maladaptive thoughts (n=8), restructuring maladaptive thoughts (n=8), attention training (n=4), and exposure to tinnitus (n=1). Besides, a larger part of the apps (n=39) informed users about the aim of the intervention, while only a few apps offered information on uncertainties and missing evidence (n=7). Across all apps, we identified 41 different intervention components. Apps highly differed in the number of intervention components they included, ranging from 2 to 20 intervention components per app. For full details on the identified intervention components, see Table 2.

**Table 2.** Intervention components used in 69 analyzed apps.^a^

Intervention component	Implemented in n apps	Key element in n apps
Assessing tinnitus characteristics	38	22
Assessing tinnitus distress	18	4
Analysis of risk factors for tinnitus	23	1
Provision of information regarding risk factors	21	
Assessment of risk factors	4	
Analysis of factors influencing the development and maintenance of tinnitus distress	16	1
Provision of information regarding influencing factors	14	
Assessment of influencing factors	4	
Information on or examination of further otological symptoms (eg, hearing loss and hyperacusis)	16	—^b^
Information on the intervention regarding^c^		
Aim of the intervention	39	—
Treatment options (including nonintervention)	1	—
Information on uncertainties and missing evidence	7	—
Probabilities of success, failure, and side effects of the intervention and of treatment options	1^d^	—
Costs	69	—
Information on tinnitus or psychoeducation	27	16
Relaxation training	16	9
Mindfulness exercises	12	3
Identification of maladaptive thoughts	8	3
Restructuring of maladaptive thoughts	8	3
Attention training	4	1
Exposure to tinnitus	1	—
Emotional regulation	1	1
Social support (eg, contact with others)	5	—
Hearing strategies for hearing impairment	2	—
Techniques to improve sleep	12	2
Techniques to improve concentration and work efficacy	2	—
Sounds (eg, white noise, nature sounds, and music)	58	52
Auditory discrimination training	1	1
Tailor-made notched music therapy	10	9
Acoustic neuromodulation	5	5
Relapse prevention	2	—
*Other* ^e^		
Behavioral activation	4	—
Resource-oriented or acceptance-based exercises^f^	4	1
Exercises focusing on residual inhibition	3	—
Tracking of tinnitus and other symptoms (eg, mood, stress, concentration, or sleep)	2	2
Exercises to improve auditory perception and directional hearing	2	1

a Intervention components were assessed using a list of typical intervention components in tinnitus treatment developed by the authors (AR, DL, and CW; Multimedia Appendix 1). Components identified in at least 1 app are depicted.

b Not applicable.

c Based on the guideline for evidence-based health information, namely requirements regarding the content of information for patients about a treatment [31].

d Developed by app community, no information provided by the app developers.

e For details on further intervention components identified in only one app, see Multimedia Appendix 1.

f For example, focus on strengths and successes, gratitude exercises, assessment of important motives and needs—setting life goals.

### Behavior Change Techniques

BCTs were categorized using the taxonomy by Michie et al [19]. In total, 36 out of 93 BCTs were identified in at least 1 app. The most frequently implemented BCTs were “instruction on how to perform the behavior” (n=25, eg, audio instructions on how to perform a relaxation technique), “feedback on behavior” (n=11), “behavioral practice/rehearsal” (n=11, eg, prompting users to practice mindfulness-based exercises), “information about health consequences” (n=11), “information about emotional consequences” (n=11), and “prompts/cues” (n=11, eg, app reminders to engage in pleasant activities). Apps highly differed in the number of implemented BCTs (none to 18 BCTs per app), with 32 apps including no BCTs at all. See Table 3 for a full overview of BCTs identified.

**Table 3.** Behavior change techniques (BCTs) identified in 69 analyzed apps^a^.

Category and BCT		Implemented in n apps
1. Goals and planning		
1.1 Goal setting (behavior)		5
1.2 Problem solving		2
1.3 Goal setting (outcome)		2
1.4 Action planning		1
1.6 Discrepancy between current behavior and goal		1
1.9 Commitment		1
2. Feedback and monitoring		
2.2 Feedback on behavior		11
2.3 Self-monitoring of behavior		2
2.4 Self-monitoring of outcome of behavior		7
2.7 Feedback on outcome of behavior		3
3. Social support		
3.1 Social support (unspecified)		4
4. Shaping knowledge		
4.1 Instruction on how to perform the behavior		25
4.3 Reattribution		1
5. Natural consequences		
5.1. Information about health consequences		11
5.4 Monitoring of emotional consequences		1
5.6 Information about emotional consequences		11
6. Comparison of behavior		
6.1 Demonstration of the behavior		3
7. Associations		
7.1 Prompts/cues		11
7.7 Exposure		1
7.8 Associative learning		1
8. Repetition and substitution		
8.1 Behavioral practice or rehearsal		11
8.3 Habit formation		2
8.7 Graded tasks		1
9. Comparison of outcomes		
9.1 Credible source		3
10. Reward and threat		
10.3 Nonspecific reward		5
10.4 Social reward		1
10.6 Nonspecific incentive		1
10.9 Self-reward		1
11. Regulation		
11.1 Pharmacological support		1
11.2 Reduce negative emotions		5
12. Antecedents		
12.1 Restructuring the physical environment		2
13. Identity		
13.2 Framing or reframing		8
13.4 Valued self-identity		2
14. Scheduled consequences		—
15. Self-belief		
15.1 Verbal persuasion about capability		1
15.3 Focus on past success		1
15.4 Self-talk		1
16. Covert learning		—

a BCTs were assessed using the behavior change technique taxonomy v1 [19]. Only those BCTs that were identified at least once are displayed.

## Discussion

### Principal Findings

In this systematic review, we assessed intervention components and BCTs [19] used in tinnitus apps and provided a detailed summary of the content of available apps. We analyzed a total of 69 apps available in English or German, all of which were easily accessible via 2 major app stores.

Within the 69 apps included in our study, numerous intervention components were identified (eg, relaxation, assessment of tinnitus distress, or techniques to improve sleep). The most frequently implemented intervention component was sounds (eg, white noise and nature sounds). Furthermore, many apps offered an assessment of tinnitus characteristics or tinnitus distress and provided information on tinnitus, risk factors, and the basics of the intervention offered (eg, its aim). One possible reason for focusing on these components is that they are relatively easy to implement. Our findings are consistent with previous research reporting the frequent use of sound therapy in tinnitus apps [14,15] and patients’ preference for such apps [18]. In a recent health service evaluation, sound therapy (including hearing aids) was found to be the most frequently available intervention in both northern and southern Europe [35]. At the same time, clinical guidelines differ on whether they recommend sound therapy, given the lack of evidence or the risk of bias in previous studies [8,36]. Although meta-analytic evidence clearly supports the efficacy of CBT for treating tinnitus distress [8,36,37], CBT intervention components (eg, cognitive restructuring, relaxation training, and attention shifting) were implemented in only a small number of apps. In contrast, when analyzing CBT apps for depression, Martinengo and colleagues identified a variety of apps offering, for example, cognitive restructuring [38]. Thus, it seems possible to realize even more complex CBT components in smartphone apps. Interestingly, a closer look at the intervention components revealed considerable differences in how certain components were implemented. For example, 1 app addressed typical maladaptive thoughts by naming several maladaptive thoughts, briefly elaborating on their potential negative effects, and suggesting alternative thoughts. In another app, users were asked to name individual debilitating thoughts and were then provided with techniques to develop a more helpful thought.

Our second aim was to assess the BCTs used in available tinnitus apps, as up-to-date knowledge on this topic is limited. In our study, we identified 36 of the 93 BCTs defined by Michie et al [19]. The 6 most frequently identified BCTs were “instruction of how to perform the behavior,” “information about emotional consequences,” “information about health consequences,” “feedback on behavior,” “behavioral practice/rehearsal,” and “prompts/cues.” Only a few other studies have assessed BCTs used in tinnitus interventions. Greenwell et al [16] analyzed BCTs in 5 studies on self-help interventions for tinnitus, and 2 studies reported on BCTs used in individual online interventions [21,22]. The techniques most frequently identified in our study are likely common because they are straightforward to implement in an app. For example, to promote relaxation behavior, users were instructed on how to perform a breathing exercise (“instruction on how to perform a behavior”), motivated to practice relaxation with audio recordings (“behavioral practice/ rehearsal”), or sent reminders to complete an exercise (“prompts/cues”). Interestingly, social support techniques were identified in only 4 out of 69 apps. This aligns with Greenwell et al [16] findings that social support components were largely absent in self-help interventions for tinnitus. In previous research on tinnitus support groups and group therapy, features such as the exchange of information, a sense of belonging, and validation of the tinnitus experience were identified as central helpful elements [39,40]. Including peer support in apps could therefore benefit tinnitus users, and smartphone apps offer various ways to provide this digitally. Moreover, goal setting was implemented in only a few apps. From a clinical perspective, prompting users to set goals and review them (“goal setting behavior,” “goal setting outcome,” “review behavior goals,” and “review outcome goals”) could enhance motivation. Reviewing these goals, according to Michie et al [19], may lead to adapting goals or modifying the behavior change strategy based on prior achievements. This process would allow users to evaluate whether they have met their goals using the app or if adjustments to the goal are needed. Additionally, normalizing the adaptation of goals or strategies may further support user motivation.

In addition to numerous apps focusing on the provision of sounds and a small number of apps offering various CBT strategies, we identified apps offering, for example, acoustic neuromodulation or tailor-made notched music therapy. This highlights the general problem that tinnitus sufferers searching for an app are confronted with treatment approaches supported by varying levels of evidence [29,37,41]. It is likely that most app users are unaware of this. Furthermore, while a few tinnitus apps have been tested for efficacy, many apps lack scientific evaluation [42-47]. This aligns with our findings from a subsample of the apps included in this study [23]. In the current study, we also found only a few apps providing information on uncertainties or missing evidence regarding the intervention provided. As a basic requirement, app developers should inform users about the evidence base of the treatment approach and whether the app has been scientifically evaluated. Additionally, information on alternative treatment approaches should be provided to enable informed decision-making. This would also provide guidance to users if they are unable to reach their goals using the app. Similarly, Sander et al [48], in their review on posttraumatic stress disorder apps, proposed that apps categorized as medical or health apps should inform users that the app is not a replacement for standard treatment and provide guidance on accessing other treatment options. Correspondingly, other groups have called for evidence-based information in medical and health-related apps [49,50]. In our subsample analysis, we identified a few intervention components that could have negative effects if used without guidance from a clinical expert (eg, insufficient instructions for a training designed for users with hyperacusis) [23]. This is consistent with previous research highlighting potential negative effects when certain exercises are used without support [18]. Remarkably, none of the apps were categorized as “barrier-free.” However, we only screened for app features aimed at reducing barriers using a single yes or no item. In light of our findings and the large number of available tinnitus apps, health care professionals should ask patients whether they are using tinnitus apps and evaluate app content together. This may help patients assess whether the interventions offered are evidence based. In addition, potential shortcomings and negative effects can be identified, and professionals can provide guidance on the proper use of certain strategies. This aligns with recommendations by Brown et al [50], who call for increased awareness of potential negative effects among users and health care professionals. Assessing apps used in daily life also offers the opportunity to integrate exercises into a broader treatment plan. For example, a patient may use relaxation training offered in an app but struggle to practice it regularly. In this case, a health care professional can help identify barriers and develop strategies to overcome them.

This review provided several key insights into the current landscape of tinnitus apps. Using a broad and systematic search strategy, we identified a large number of tinnitus-specific apps and evaluated each app in detail, rather than focusing only on the most popular ones. This approach allowed us to provide a comprehensive overview of the intervention components and BCTs currently implemented in tinnitus apps. A key strength of this review is the high sensitivity of the initial search. By using a broad search strategy, we accounted for nonclinical terminology often used by developers in app store metadata. During the screening phase, a significant number of hits had to be excluded because the apps were not related to tinnitus (eg, offering a decibel meter). Probably due to the app store search algorithms using a multitude of criteria (eg, screening title, description, and keywords), 153 apps (eg, accompanying hearing aid devices or generic sound or relaxation apps that lacked tinnitus-specific content) were further retrieved. While these apps might have used “tinnitus relief” as a keyword in their metadata to, for example, increase visibility, they were not specifically designed for tinnitus and were, therefore, excluded from the final analysis. Although the broad search strategy resulted in a high exclusion rate of apps, it ensured that no dedicated tinnitus-specific interventions were overlooked, providing a more reliable overview of the current digital health landscape for tinnitus.

Our findings show that sounds were by far the most frequently implemented intervention component, whereas evidence-based CBT components were present in only a small proportion of apps, despite strong evidence for CBT in the treatment of tinnitus distress. Furthermore, only 36 of the 93 BCTs defined by Michie et al [19] were identified across all apps, indicating a limited use of established BCTs. By systematically mapping these components, we highlight which BCTs are currently under-represented and discuss their potential relevance for future app development. In addition, our results underscore the lack of transparent information on the evidence base of the treatment approaches offered in many apps and the need for scientific evaluation. Regular assessment of tinnitus apps by health care professionals may help identify potential shortcomings, support informed use of apps, and facilitate their integration into a broader treatment plan.

### Limitations and Future Directions

Besides these strengths, several limitations should be considered. First, the systematic search was last updated in 2023. Accordingly, newly developed apps may not be included in the analysis. Furthermore, some of the included apps may no longer be available on the app stores, or their content might have changed substantially since the time of data extraction, limiting the temporal validity of our findings. Moreover, more recently developed apps might include more complex CBT components or a greater variety of BCTs. We searched 2 major app stores (Google Play Store and Apple App Store) but did not search app libraries (eg, ORCHA App Library) or other app stores (eg, Windows Store). Thus, the coverage of available apps may be incomplete. It is also possible that additional tinnitus apps were unavailable on the German app store but accessible to users in other countries. Second, we only analyzed apps available in English or German. Therefore, our findings may not be generalizable to apps in other languages and potential cultural or regional differences in app design and content are not captured. Third, as we used tinnitus-specific search terms and focused solely on apps explicitly developed for tinnitus, this review does not cover other apps that might potentially be beneficial for people suffering from tinnitus. In particular, transdiagnostic or mental health apps (eg, targeting stress or sleep) that may indirectly address tinnitus-related distress were not included, which may limit the scope of the review. Fourth, although we provide an overview of intervention components currently used in apps, no conclusions about the quality of their digital implementation can be drawn based on our data. Accordingly, we did not assess the mode of delivery for individual BCTs or how often they were used in each app. Our coding was based on the presence or absence of components rather than their depth or user engagement, which may vary substantially between apps. Fifth, based on our results, we cannot investigate associations between the implementation of a particular BCT or intervention component and the efficacy of an app. We did not incorporate user-level data (eg, adherence, engagement, or outcomes), which limits conclusions regarding the practical effectiveness of the apps and the identified components.

Future research is needed to determine which intervention components, BCTs, or combination of BCTs are most effective in promoting behavior change and reducing tinnitus-related distress, ideally by linking specific components to user engagement and clinical outcomes. Moreover, subsequent studies may clarify how complex CBT components, such as cognitive restructuring, can be realized in an app to be beneficial for the user. Such research should also include questions of usability, personalization, and appropriate guidance. Future research should also examine how intervention components need to be implemented to be used safely without professional supervision, and under which conditions additional human support may be required to ensure both safety and effectiveness.

### Conclusions

In this review, we provide a detailed and systematic analysis of tinnitus app content by categorizing intervention components and BCTs. By applying an established BCT framework, this study offers a structured and theory-informed overview of current app-based interventions. A variety of intervention components were identified at least once (eg, relaxation training, sound therapy, and information on tinnitus). However, the component most frequently implemented was sounds (eg, white noise and nature sounds), highlighting a discrepancy between commonly implemented features and evidence-based treatment recommendations. Core CBT elements, which are recommended in tinnitus treatment guidelines, were offered less frequently. Patients with tinnitus searching for support in app stores are confronted with a large number of easily accessible apps, yet the content and theoretical grounding of these apps vary substantially. In light of the limited scientific evaluation of these apps, future research on their efficacy as well as potential negative effects is highly needed. Importantly, this review provides a transparent and reproducible foundation for such evaluations by systematically mapping existing intervention components and BCTs. Overall, this study provides an overview for research and practice by summarizing current tinnitus app content and identifying key gaps that can inform evidence-based app development and future research priorities.

## Supplementary material

10.2196/66151 Multimedia Appendix 1 List of typical intervention components in tinnitus treatment, list of apps analyzed and further intervention components identified.

## Abbreviations

BCT: behavior change technique
CBT: cognitive behavioral therapy
IMI: internet- and mobile-based intervention
MARS-G: German version of the Mobile App Rating Scale
MARS-G: German version of the Mobile App Rating Scale
SPSS: Statistical Package for the Social Sciences
