# Supplementary material for: Mobile Apps for Tinnitus: Systematic Search in App Stores and Review of Intervention Components and Behavior Change Techniques
Source: JMIR Mhealth Uhealth. 2026 May 19;14:e66151. doi: 10.2196/66151 (PMC13186522; doi:10.2196/66151)
Supplement: Multimedia Appendix 1 [file mhealth-v14-e66151-s001.docx]

**Multimedia Appendix 1:**

List of typical intervention components in tinnitus treatment developed by the authors (AR, DL, CW)

| **Intervention component** |
| --- |
| Assessing tinnitus characteristics |
| Assessing tinnitus distress |
| Analysis of risk factors for tinnitus |
| Provision of information regarding risk factors |
| Assessment of risk factors |
| Analysis of factors influencing the development and maintenance of tinnitus distress |
| Provision of information regarding influencing factors |
| Assessment of influencing factors |
| Information on/ Examination of further otological symptoms (e.g. hearing loss, hyperacusis) |
| Information on the intervention regarding |
| Aim of the intervention |
| Prognosis in case of non-intervention |
| Treatment options (including non-intervention) |
| Information on uncertainties and missing evidence |
| Probabilities of success, failure and side effects of the intervention and of  treatment options |
| Costs |
| Information on tinnitus/ psychoeducation |
| Relaxation training |
| Mindfulness exercises |
| Identification of maladaptive thoughts |
| Restructuring of maladaptive thoughts |
| Attention training |
| Exposure to tinnitus |
| Exposure to avoided daily live situations or activities |
| Emotion regulation |
| Social support (e.g. contact with others) |
| Social skills training |
| Hearing strategies for hearing impairment |
| Techniques to improve sleep |
| Techniques to improve concentration and work efficacy |
| Sounds (e.g. white noise, nature sounds, music) |
| Auditory discrimination training |
| Tailor-made notched music therapy |
| Acoustic neuromodulation |
| Biofeedback therapy |
| Neurofeedback therapy |
| Relapse prevention |
| *Other* |

**Multimedia Appendix 2:**

List of all apps included in the final analysis (*n* = 69).

| **App** | **Developer** |
| --- | --- |
| Tinnitus Tailor | Glasgow Neuro LTD |
| My Practical Tinnitus | Robert Redfern |
| T-Mask | Erik Dejonckheere |
| Tinnitus: relief for your ears | Pierre Stanislas |
| Dr Mollin Tinnitus | Magdalena Gorecka |
| Beyond Tinnitus Sound | Mind:Set Technologies |
| SoundRelief by Lios | Restored Hearing Ltd |
| Shades of Noise | Tinnitus Research Initiative Foundation |
| TinniTrain | InnoApp GbR |
| Relax Noise 3 - Tinnitus | Martin Nathansen |
| Tinnitus Sound Therapy | RelaxingSmoothingMusic |
| White Noise : Tinnitus Relief | RelaxingSmoothingMusic |
| Tinnitus Noiser Cricket | ChriDator |
| Tinnitus Therapy Pro | spikything.com |
| Tinnibot: tinnitus self-care | Hearing Power |
| Tinnitus Notched Tunes | Doc Marten |
| PerNitus | WonderUX |
| Tinnitus Relief | Develant Technologies AB |
| Whist - Tinnitus Relief | Sensimetrics Corporation |
| White Noise for Sleep. Generator for Tinnitus | Maxpir Music and Sounds |
| H & T Sound Therapy | Ileisou |
| Tinnitus Relief App. Klangtherapie | Limoni Audio Sounds |
| Tonal Tinnitus Therapy | appyhapps.nl |
| Tinnitus Sound Therapy | soundsofthesoul |
| Tinnitus Hyperacusis Pro | Jined |
| Tinnitus Tuner | narusat |
| Tinnitracks Tinnitus Therapie | Sonormed GmbH |
| ReSound Tinnitus Relief | GN Resound A/S |
| Tinnitus Free | VegaMedia |
| Tinnitus DE | John goodstadt |
| Widex Zen, Tinnitus Management | Widex A/S |
| Kalmeda Tinnitus-App | mynoise GmbH |
| tinnitus help | IND-Ingenieurbuero f. Nachrichten- u. Datentechnik |
| AudioNotch | AudioNotch, LLC |
| Tinnitus HQ | Phase4 Mobile, Inc. |
| Track Your Tinnitus | Tinnitus Research Initiative Foundation |
| Tinnitus Behandlung | Rehegoo Sp. ZOO |
| Beltone Tinnitus Calmer | GN Hearing Care Corporation |
| Diapason para tinnitus | Immersive Therapy |
| Tinnitus Angel Sound Therapies | JX Labs Limited |
| Starkey Relax | Starkey Laboratories, Inc. |
| Tinnitus Therapy Pro | Headwaters Inc. / Sound-Oasis |
| Tunnitus Describer | Isam Al Saadi |
| Quieten : Tinnitus Relief | Julian Cowan Hill |
| Tinnitus Play | GLOBAL INTERACTIVE MARKETING ON-LINE LIMITED |
| T-Minus - Tinnitus Wellness | Abbey Records Ltd / T-Minus |
| Tinnitus Masker | BarraxWorld Ltd |
| TINNITUS, WHITE NOISE, SCHLAF | Alexander Bredikhin |
| Oto: CBT for Tinnitus | Oto Health Ltd |
| UNITI Tinnitus Studie | Tinnitus Research Initiative Foundation |
| Beltone Tinnitus Relief Music | Dharmesh Malaviya |
| GaertnerKlinik | HNO Klinik Dr. Gaertner |
| Harmody | Tech & Life Solutions GmbH |
| Meine Tinnitus App | Sonormed |
| Pauze your tinnitus | Immersive Therapy |
| Siopi | Siopi |
| Sound Oasis S-6000 | Sound Oasis |
| TINNITUS, WHITE NOISE | Dimitri Kirkhanidi |
| TinAid - Tinnitus Masker | Appipe |
| Tinnitus App by Cleanhearing | Cleanhearing K. K. |
| Tinnitus Compass | Kog Kon GmbH |
| Tinnitus Relief Sound | Josue Montano |
| Tonal Therapy | Fickle Bits, LLC |
| Tinnitus-Klangtherapie | Enpower Apps |
| TiFi - Tinnitus music filter | STSAM |
| Tinnitus - Relief & Therapy | VBApps |
| Tinnitus Therapy | Microsys Com Ltd. |
| Tinnitus Therapy Sound | DT productions |
| Mehrtongenerator | OK soft |

**Multimedia Appendix 3:**

Further intervention components identified in *n* = 1 app: a) stress-management, b) exercise, physical therapy, c) craniosacral therapy, d) associative learning, key component, e) amplitude modulated noise, key component, f) “habituation therapy”, app builds a therapy tone based on tinnitus assessment, key component, g) tinnitus-centered music therapy, key component, h) general advice on ear hygiene, i) analysis of influencing factors on the tinnitus tone, j) diary entries, k) dietary changes.
